# Dietary patterns, *BCMO1* polymorphisms, and primary lung cancer risk in a Han Chinese population: a case-control study in Southeast China

**DOI:** 10.1186/s12885-018-4361-2

**Published:** 2018-04-19

**Authors:** Fei He, Ren-dong Xiao, Tao Lin, Wei-min Xiong, Qiu-ping Xu, Xu Li, Zhi-qiang Liu, Bao-chang He, Zhi-jian Hu, Lin Cai

**Affiliations:** 1 Department of Epidemiology, School of Public Health, Fujian Medical University, Fuzhou, 350108 China; 2 Key Laboratory of Ministry of Education for Gastrointestinal Cancer, Fujian Medical University, Fuzhou, 350108 China; 3 Department of Thoracic Surgery, The first affiliated hospital of Fujian Medical University, Fuzhou, 350005 China

**Keywords:** Dietary patterns, *BCMO1* polymorphisms, Primary lung cancer, Chinese, Case-control study

## Abstract

**Background:**

We investigated whether *BCMO1* variants and dietary patterns are associated with lung cancer risk.

**Methods:**

Case-control study including 1166 lung cancer cases and 1179 frequency matched controls was conducted for three *BCMO1* variants (rs6564851, rs12934922, and rs7501331) and four dietary patterns were investigated. Logistic regression was used to estimate odds ratios (ORs) and 95% confidence intervals (95% CIs).

**Results:**

The rs6564851, rs12934922, and rs7501331 were not found to be associated with lung cancer risk (*P* > 0.05). In multivariable-adjusted models, compared to the lowest quartile of the score on the “fruits and vegetables” pattern, the highest quintile was associated with a 78.4% decreased risk (OR _Q4 vs. Q1_ = 0.216; 95% CI, 0.164–0.284; P for trend < 0.001). Other patterns were not found the association. The “fruits and vegetables” pattern was associated with a reduced risk of lung cancer with all 3 SNPs irrespective of genotypes (all P for trend< 0.001). The association for the “Frugal” pattern was associated with increased risk of lung cancer among smokers (P for interaction = 0.005). The protective effects of the “cereals/wheat and meat” pattern was more evident for squamous cell carcinoma and other histological type.

**Conclusions:**

We did not observe associations of *BCMO1* variants and lung cancer. Diets rich in fruits and vegetables may be protective against lung cancer.

**Electronic supplementary material:**

The online version of this article (10.1186/s12885-018-4361-2) contains supplementary material, which is available to authorized users.

## Background

The International Agency for Research on Cancer reported a lung cancer incidence rate of 23.1/100,000 and a lung cancer mortality rate of 19.7/100,000 for 2012 [1]. Although China does not yet have a well-established cancer registry system, the data available for 2015 indicate that lung cancer is the most common and most deadly cancer in China [2]. Because of the poor prognosis and often aggressive nature of lung cancer, the 5-year overall survival rate for lung cancer is only 10–15%, putting a heavy burden on patients, patient’s families, and governments [3]. Although tobacco smoking is the most salient cause of lung cancer, several other risk factors may contribute to the disease [4, 5].

It has been reported that about a third of all tumors may related to dietary factors [6]. Currently, most studies that have examined the influence of dietary factors on lung cancer risk have focused on a single food or a limited combination of certain foods or nutrients, and their results have not been consistent [7–10]. However, generally, people do not consume single foods or nutrients. Moreover, different categories of foods and nutrients may have interactions with one another. Hence, exploring specific foods and nutrients in isolation is not representative of real-life diets. Consequently, researchers have become interested in examining the influence of dietary patterns and holistic dietary status on lung cancer risk [11–18]. Although the findings are uncertain, they argue that a diet with high vegetable is related with a reduced risk of lung cancer [19], while a high fat and red meat diet is related with increased risk. However, most of these studies were conducted outside of China, where eating habit vary greatly across different regions. Examination of the possible influence of Chinese dietary patterns on lung cancer is lacking.

Importantly, any observed correlation between dietary patterns and lung cancer could be related to other factors, such as smoking, social-economic status, and physical activity [19]. Although intake of vegetables and fruits has been suggested to reduce the risk of lung cancer [10], β-carotene (BC)—a retinal (form of vitamin A) precursor found in many edible plants—has been suggested to increase the risk of lung cancer in smokers [7], perhaps due to a complex gene-diet interaction. In this regard, Tu and colleagues suggested recently that the association between dietary patterns and lung cancer risk may be modified by genetic background [17].

Dietary BC is cleaved into two retinal molecules by β-carotene-15,15′-monooxygenase (BCMO1) [20]. Single nucleotide morphisms (SNPs) of the human *BCMO1* gene, which is located on chromosome 16, have been reported to influence blood concentrations of BC, suggesting that *BCMO1* SNPs may affect the efficiency of BC transformation into vitamin A in vivo [21]. If so, then it is possible that *BCMO1* SNPs may also influence the effects of dietary patterns on lung cancer risk. To test this hypothesis, we conducted a case-control study to explore the potential influence of three *BCMO1* SNPs, namely rs6564851, rs12934922, and rs7501331, on the association between dietary patterns and lung cancer risk in a case-control study of ethnic Han Chinese participants.

## Methods

### Study subjects

We recruited 1166 patients with newly diagnosed (the time of cancer diagnosis and the time of enrolling into the study was the same) primary lung cancer (cases) from three area hospitals (The First Clinical Medical College of Fujian Medical University, The Affiliated Union Hospital of Fujian Medical University and Fuzhou General Hospital) between July 2006 and February 2013 and the participate rate for patients was 96.20%. The non-responders included 32 male and 14 female, average age was 58.93 ± 15.44 years, so there were no differences between the responders and non-responders. One thousand one hundred seventy-nine gender- and age-matched healthy controls (±2 years) randomly selected from the community between July 2006 and February 2013. Individuals who were direct relatives to the cases or had a previous history of cancer were excluded. The rate for control subjects was 90.01%. The non-responders included 92 male and 39 female, average age was 59.66 ± 12.17 years, so there were no differences between the responders and non-responders. All cases and controls were Fujian Province residents. This study was approved by the Institutional Review Board of Fujian Medical University (Fuzhou, China) and all participants signed informed consent forms ([2014] Fu Yi Ethics Review (No. 98)).

### Data collection

All epidemiological data were obtained by in-person interviews with a standardized questionnaire, which collected information on demographic characteristics, disease and family cancer history, food, tobacco use, tea and wine consumption, environmental tobacco exposure. Using the inquiry method for surveying dietary habits, respondents recalled their average frequency of consumption of foods (grams per day) in last year (the year prior to study enrolment for all objects) for a variety food items including cereals/wheat, potatoes, meat (pork, beef, mutton, poultry), eggs, seafood (fish, shellfish, snails, salted fish), kelp and seaweed, beans (soy products, dried beans), milk, fruits, vegetables, salted vegetables. The questionnaire has been shown to be a valid and reliable food frequency survey tool across various populations [22–24].

Smokers were defined as individuals who had smoked at least 100 cigarettes during their lifetime. Environmental tobacco smoke (ETS) was defined as exposure to ETS at home and/or at work for more than 15 min per day. Drinking alcohol was defined as drinking at least once a week for more than half a year. Drinking tea was defined as drinking at least 1 cup a week for more than half a year. A 5-ml non-fasting blood sample was collected from each participant for genotyping.

### Selection of SNPs

We selected three common (minor allele frequency > 5%) SNPs for analysis, namely rs6564851, rs12934922, and rs7501331. Two of these (rs12934922 and rs7501331) are non-synonymous mutations, identified as yielding a 57% reduction in the catalytic activity of BCMO1 (*P* < 0.001) [25]. The third SNP, rs6564851, was identified by a genome-wide association study, wherein it was associated with elevated plasma β-carotene and low plasma lutein [26].

### Genotyping

Genomic DNA was extracted from the blood samples with a protease K digestion and phenol-chloroform extraction and purification system according to standard procedures. The genomic DNA was stored at − 20 °C until being subjected to SNP genotyping with the Sequenom platform according to the manufacturer’s iPLEX Application Guide (Sequenom, Inc., San Diego, CA). The samples were scanned through a matrix assisted laser desorption ionization-time of flight mass spectrometry system and genotyped with a MassArrayTyper 3.4 (Sequenom Inc. San Diego, CA). Approximately 10% of the samples (randomly selected) were re-run for quality control purposes. Genotyping call rates were > 90% and the concordance rate reached 99.5%.

### Statistical methods

Descriptive statistics were performed to characterize the study subjects. In the preliminary stage of statistical analysis, the chi-square test was employed to examine differences in demographic variables between cases and controls.

We identified dietary patterns using principal components factor analysis based on responses to the baseline questionnaire. We designated 11 food items. Using the food frequency survey, we collected information about the types and quantities of dietary intake from all subjects for the past year (i.e., the 12 months before the survey was administered). We standardized the quantity values to a mean of 0 and a standard deviation (SD) of 1.0. Each of the standardized quantity variables were entered in the factor analysis; based on inspection of scree plots, eight factors were retained. The factors were rotated using the quartimax procedure to facilitate interpretability of the factors. Factor scores were categorized into quartiles based on the sex-specific distribution in the control group.

The associations between each factor and the risk of lung cancer were estimated by calculating the crude and adjusted odds ratio (OR) for confounders and a 95% confidence interval (CI) with unconditional logistic regressions for factor scores on each of the four factors, the multivariate models adjusted for potential confounders based on a priori knowledge. And we also investigated the associations between dietary patterns and lung cancer risk striated by smoking status and SNP of interest. To detect trends, we entered the factor scores into the model as continuous terms. A two-tailed *p*-value less than 0.05 was considered to be statistically significant. All statistical analyses were performed in the R software package (v. 3.3.1).

## Results

### Characteristics of study subjects

The demographic characteristics and risk factors for cases (*N* = 1166) and controls (*N* = 1179) are summarized in Table 1. A lower BMI (*P* < 0.001), lower income (*P* = 0.031), tobacco smoking (OR = 2.451; 95% CI, 2.075–2.894), and ETS exposure (OR = 2.859; 95% CI, 2.412–3.388), together with family cancer history (OR = 1.373, 95% CI, 1.105–1.706) and lung disease history (OR = 1.697; 95% CI, 1.301–2.214) were associated with lung cancer. In contrast, a high educational background emerged as a protective factor against lung cancer (*P* < 0.001). Additionally, different occupations had different associated risks for lung cancer (*P* < 0.001).

**Table 1:** Distribution of selected variables among cases and controls

Variables	Case (%)	Control (%)	*P* value	OR (95% CI)
	(*n* = 1166)	(*n* = 1179)		
Age (yrs.) mean ± SD	58.28 ± 11.28	59.19 ± 10.78	0.149	
< 50	223(19.1)	264(22.4)		1
51–69	739(63.4)	716(60.7)		1.222 (0.995–1.501)
≥ 70	204(17.5)	199(16.9)		1.214 (0.932–1.581)
Income(yuan/month)				
≤ 3500	797 (67.6)	836 (71.7)	0.031	1
> 3500	382 (32.4)	330 (28.3)		0.824 (0.690–0.982)
Gender			0.542	
Male	842 (72.2)	838 (71.1)		1
Female	324 (27.8)	341 (28.9)		0.946 (0.790–1.132)
Education			< 0.001	
Illiteracy	185(15.9)	133(11.3)		1
Middle school and below	686(58.8)	647(54.9)		0.762 (0.595–0.976)
High school and above	295(25.3)	399(33.8)		0.532 (0.406–0.696)
Marital status			0.15	
Married	1099(94.3)	1094(92.8)		1
Single	67(5.7)	85(7.2)		0.785(0.564–1.092)
Occupation			< 0.001	
Worker	266(22.8)	297(25.2)		1
Farmer	333(28.6)	245(20.8)		1.518 (1.201–1.917)
Enterprises and employees	321(27.5)	433(36.7)		0.828 (0.665–1.031)
Cook	18(1.5)	9(0.8)		2.233 (0.986–5.055)
Others	228(19.6)	195(16.5)		1.305 (1.014–1.681)
Family history of lung cancer			0.004	
No	942(80.8)	1005(85.2)		1
Yes	224(19.2)	174(14.6)		1.373(1.105–1.706)
History of lung diseases			< 0.001	
No	1009(86.5)	1080(91.6)		1
Yes	157(13.5)	99(8.4)		1.697(1.301–2.214)
BMI (kg/m^2^)			< 0.001	
18.5–23.9	720(61.9)	638(54.5)		1
< 18.5	133(11.4)	51(4.4)		2.311(1.645–3.246)
≥ 24	311(26.7)	481(41.1)		0.573(0.479–0.685)
Tea			0.729	
No	578(49.6)	576(48.9)		1
Yes	588(50.4)	603(51.1)		0.972(0.826–1.143)
Alcohol			0.353	
No	884(75.8)	913(77.4)		1
Yes	282(24.2)	266(22.6)		1.095(0.904–1.326)
Smoking			< 0.001	
No	427(36.6)	691(58.6)		1
Yes	739(63.4)	488(41.4)		2.451(2.075–2.894)
ETS			< 0.001	
No	348(29.8)	647(54.9)		1
Yes	818(70.2)	532(45.1)		2.859(2.412–3.388)
Histology				
Adenocarcinoma	551(47.3)			
Squamous cell carcinoma	324(27.8)			
Others	290(24.9)			

### SNP effects on lung cancer risk

The genotype frequencies for all three SNPs examined conformed to the Hardy-Weinberg equilibrium (HWE) in the control group (*P*_*controls*_ = 0.09–0.88). The genotype frequency data for these SNPs in the case and control groups are reported in Table 2 with the corresponding ORs for lung cancer. Neither the rs6564851, rs12934922, nor rs7501331 variant genotypes of *BCMO1* were found to be associated with lung cancer risk, with or without controlling for the effects of potentially confounding factors.

**Table 2:** Distribution of *BCMO1* single nucleotide polymorphisms and their associations with lung cancer

Locus	Case (*n* = 1166)	Control (*n* = 1179)	Unadjusted OR	Adjusted OR*	*P*_*trend*_ value
			95% CI	95% CI	
rs6564851(*P*_HWE_ = 0.57)	1097	1102			0.729
GG	743 (67.7%)	724 (65.7%)	1	1	
GT	310 (28.3%)	342 (31.0%)	0.883 (0.734–1.062)	0.922 (0.754–1.128)	
TT	44 (4.0%)	36 (3.3%)	1.191 (0.758–1.872)	1.311 (0.805–2.134)	
GT + TT	354 (32.3%)	378 (34.3%)	0.913 (0.764–1.090)	0.959 (0.791–1.163)	
rs12934922(*P*_HWE_ = 0.88)	1121	1146			0.672
AA	839 (74.8%)	861 (75.1%)	1	1	
AT	261 (23.3%)	264 (23.0%)	1.015 (0.834–1.234)	0.955 (0.772–1.182)	
TT	21 (1.9%)	21 (1.8%)	1.026 (0.556–1.893)	0.813 (0.424–1.561)	
AT+TT	282 (25.2%)	285 (24.8%)	1.015 (0.840–1.228)	0.944 (0.768–1.160)	
rs7501331(*P*_HWE_ = 0.09)	1060	1084			0.117
CC	707 (66.7%)	696 (64.2%)	1	1	
CT	308 (29.1%)	334 (30.8%)	0.908 (0.753–1.094)	0.862 (0.704–1.056)	
TT	45 (4.2%)	54 (5.0%)	0.820 (0.545–1.235)	0.829 (0.531–1.294)	
CT + TT	353 (33.3%)	388 (35.8%)	0.896 (0.749–1.070)	0.858 (0.707–1.041)	

*adjusted by incomes, occupation, education, family history of lung cancer, history of lung diseases, environmental tobacco smoke, smoking status, BMI

### Dietary patterns analysis

Before rotation, the four primary dietary pattern factors identified by our principal components factor analysis explained 49.53% of the variance in cases and controls. The foods and factor weightings for each factor are shown in Additional file 1: Table S1. For the first factor, the highest factor weight was concentrated in high quality protein, such as seafood, kelp and seaweed, egg and beans. The second factor was milk, fruits and vegetables. The most heavily weighted foods in the third factor were traditional pattern, including cereals/wheat and meat. Sweet potato and salty vegetables were the highest weighted contributors to the fourth factor. The four dietary patterns were named “high quality protein”, “fruits and vegetables”, “cereals/wheat and meat” and “frugal pattern”. All patterns complied with the dietary characteristic and traditions of the Fujian people in China, indicating that the factors captured distinct sources of local dietary variation.

### Baseline characteristics of all subjects by quartile (Q) of factor score

The characteristics of the individuals associated with each of the four dietary patterns are summarized in Additional file 2: Table S2. Relative to the other participants, people with a sea food-dominant diet (high quality protein pattern) were younger, were more likely to be college graduates, exposure to less ETS and consumed more tea. Meanwhile, those with the fruits and vegetables pattern were associated with higher education background, and to have more frequent exposure to smoking, increased tea intake, and decreased ETS. High scores for cereals/wheat and meat pattern were younger, with adenocarcinoma, more common in female than male and were associated with family history of lung cancer, decreased tobacco and tea use, and increased ETS. The frugal pattern was associated with a lower education level and income, and greater ETS exposure.

### Associations of dietary pattern and lung cancer risk

Multivariable-adjusted associations of dietary patterns with lung cancer risk are presented in Table 3. In multivariable-adjusted models, compared to the lowest quartile of the score on the “fruits and vegetables” pattern, the highest quintile was associated with a 78.4% decreased risk and dose-response relationship (OR _Q4_ vs. _Q1_ = 0.216; 95% CI, 0.164–0.284; P for trend < 0.001). Other patterns were not found the association. The stratified associations by histological type of lung cancer is also summarized in Table 3. The “fruits and vegetables” pattern was associated with risks of all histological types. The protective effects of the “cereals/wheat and meat” pattern was more evident for squamous cell carcinoma and other histological type.

**Table 3:** Associations between dietary patterns by quartile (Q) and lung cancer risk by histological types

Dietary pattern	Controls	Adenocarcinoma		Squamous cell carcinoma		Others		All	
		N	adjusted OR*(95% CI)	N	adjusted OR*(95% CI)	N	adjusted OR*(95% CI)	N	adjusted OR*(95% CI)
High quality protein									
Q1(low)	294	126	1	74	1	77	1	277	1
Q2	296	130	1.119 (0.824–1.520)	85	1.250 (0.856–1.824)	75	1.068 (0.735–1.552)	290	1.141 (0.891–1.460)
Q3	295	142	1.196 (0.883–1.618)	88	1.341 (0.920–1.954)	62	0.906 (0.615–1.336)	292	1.134 (0.885–1.452)
Q4(high)	294	153	1.400 (0.999–1.890)	77	1.254 (0.853–1.845)	76	1.192 (0.820–1.734)	306	1.283 (0.999–1.643)
P for trend			0.170		0.406		0.785		0.063
Fruits and vegetables									
Q1(low)	294	283	1	160	1	160	1	603	1
Q2	295	121	0.439 (0.332–0.581)	79	0.513 (0.365–0.720)	61	0.398 (0.280–0.565)	261	0.447 (0.354–0.566)
Q3	296	85	0.282 (0.208–0.384)	51	0.330 (0.225–0.484)	40	0.247 (0.166–0.369)	176	0.285 (0.221–0.368)
Q4(high)	294	62	0.213 (0.152–0.298)	34	0.235 (0.152–0.363)	29	0.188 (0.120–0.295)	125	0.216 (0.164–0.284)
P for trend			< 0.001		< 0.001		< 0.001		< 0.001
Cereals/wheat and meat									
Q1(low)	294	117	1	92	1	84	1	293	1
Q2	295	146	1.149 (0.847–1.561)	93	0.848 (0.592–1.215)	78	0.820 (0.569–1.182)	317	0.973 (0.759–1.246)
Q3	296	127	0.920 (0.673–1.257)	83	0.816 (0.565–1.179)	70	0.744 (0.512–1.081)	280	0.846 (0.658–1.086)
Q4(high)	294	164	1.179 (0.871–1.597)	56	0.534 (0.358–0.796)	58	0.588 (0.397–0.872)	278	0.831 (0.645–1.070)
P for trend			0.342		0.012		0.021		0.230
Frugal pattern									
Q1(low)	294	126	1	75	1	63	1	264	1
Q2	296	117	0.857 (0.627–1.171)	72	0.841 (0.569–1.243)	66	0.951 (0.638–1.417)	255	0.873 (0.675–1.129)
Q3	295	118	0.827 (0.605–1.131)	81	0.894 (0.610–1.311)	73	1.022 (0.690–1.514)	272	0.897 (0.695–1.159)
Q4(high)	294	190	1.337 (0.998–1.790)	96	1.029 (0.710–1.491)	88	1.216 (0.831–1.779)	374	1.235(0.966–1.581)
P for trend			0.050		0.791		0.267		0.073

*adjusted by incomes, occupation, education, family history of lung cancer, history of lung diseases, environmental tobacco smoke, smoking status, BMI

### Stratified associations by smoking status

The negative association of the “fruits and vegetables” pattern with lung cancer risk was present among never or smokers, and the P for interaction was 0.002. The “Cereals/wheat and meat” pattern was associated with an increased risk of lung cancer among never smokers and a decreased risk of lung cancer among smokers, with the P for interaction (< 0.001) was statistically significant. The association for the “Frugal” pattern was associated with increased risk of lung cancer among smokers (P for interaction = 0.005). The association for the “High quality protein” pattern did not differ by smoking status (P for interaction = 0.570) (Table 4).

**Table 4:** Associations between dietary patterns by quartile (Q) and lung cancer risk by smoking status

Dietary pattern	Never smokers		Smokers	
	Cases/controls	Adjusted OR*(95% CI)	Cases/controls	Adjusted OR*(95% CI)
High quality protein				
Q1(low)	104/172	1	173/122	1
Q2	102/177	1.085 (0.753–1.563)	189/119	1.226 (0.865–1.737)
Q3	108/169	1.244 (0.864–1.790)	184/126	1.151 (0.813–1.631)
Q4(high)	113/173	1.397 (0.972–2.008)	193/121	1.310 (0.924–1.857)
P for trend		0.053		0.187
Fruits and vegetables				
Q1(low)	239/154	1	364/140	1
Q2	88/165	0.365 (0.258–0.517)	173/130	0.529 (0.383–0.730)
Q3	53/184	0.180 (0.121–0.266)	123/112	0.410 (0.289–0.580)
Q4(high)	47/188	0.159 (0.106–0.239)	79/106	0.270 (0.184–0.395)
P for trend		< 0.001		< 0.001
Cereals/wheat and meat				
Q1(low)	85/189	1	208/105	1
Q2	82/167	1.136 (0.774–1.666)	229/128	0.827 (0.588–1.164)
Q3	120/168	1.351 (0.935–1.951)	161/128	0.556 (0.392–0.794)
Q4(high)	137/167	1.517 (1.053–2.184)	141/127	0.463 (0.322–0.666)
P for trend		0.016		< 0.001
Frugal pattern				
Q1(low)	117/163	1	147/131	1
Q2	94/176	0.661 (0.458–0.956)	161/120	1.142 (0.799–1.633)
Q3	90/177	0.620(0.428–0.898)	182/118	1.278 (0.895–1.824)
Q4(high)	126/175	0.865 (0.607–1.232)	249/119	1.713 (1.215–2.416)
P for trend		0.436		0.002

*adjusted by incomes, occupation, education, family history of lung cancer, history of lung diseases, ETS and BMI

### Stratified associations by BCMO1 loci

The stratified associations of dietary patterns with lung cancer risk by *BCMO1* genotype at 3 SNPs are summarized in Table 5. The “fruits and vegetables” pattern was associated with a reduced risk of lung cancer with all 3 SNPs irrespective of genotypes and a dose-response relationship (all P for trend< 0.001). In contrast, the “High quality protein” pattern was associated with an increased risk of lung cancer only among those with one copy of the minor allele of rs6564851 (OR _Q4 vs. Q1_ = 1.870; 95% CI,1.206–2.900; P for trend = 0.001; P for interaction = 0.019). The “Frugal” pattern was associated with an increased risk of lung cancer among those with the wild genotype at rs6564851 and rs7501331 (P for trend < 0.05). No statistically significant were found between “Cereals/wheat and meat” patterns and all 3 SNPs (Table 5).

**Table 5:** Associations between dietary patterns and lung cancer risk by genotype at 3 SNPs

Dietary patterns	Cases/controls	Adjusted OR (95% CI)	Cases/controls	Adjusted OR (95% CI)	P for interaction
	rs6564851:GG		rs6564851:GT + TT		
High quality protein		*P*_*trend*_ = 0.655		*P*_*trend*_ = 0.001	0.019
Q1(low)	186/169	1.00(ref)	80/111	1.00(ref)	
Q2	195/180	1.112 (0.807–1.531)	76/99	1.186 (0.758–1.855)	
Q3	176/184	0.999 (0.722–1.381)	97/82	1.869 (1.200–2.910)	
Q4(high)	186/191	1.120 (0.811–1.547)	98/86	1.870 (1.206–2.900)	
Fruits and vegetables		*P*_*trend*_ < 0.001		*P*_*trend*_ < 0.001	0.792
Q1(low)	386/186	1.00(ref)	181/91	1.00(ref)	
Q2	162/182	0.413 (0.304–0.559)	82/94	0.511 (0.339–0.770)	
Q3	114/176	0.300 (0.216–0.416)	53/99	0.280 (0.179–0.437)	
Q4(high)	81/180	0.217 (0.153–0.309)	38/94	0.227 (0.140–0.367)	
Cereals and meat		*P*_*trend*_ = 0.036		*P*_*trend*_ = 0.541	0.1
Q1(low)	188/165	1.00(ref)	89/119	1.00(ref)	
Q2	203/191	0.857 (0.622–1.181)	91/85	1.230 (0.797–1.899)	
Q3	170/185	0.726 (0.523–1.008)	92/89	1.163 (0.757–1.787)	
Q4(high)	182/183	0.729 (0.525–1.012)	82/85	1.163 (0.748–1.809)	
Frugal pattern		*P*_*trend*_ = 0.028		*P*_*trend*_ = 0.445	0.371
Q1(low)	165/192	1.00(ref)	83/86	1.00(ref)	
Q2	173/183	1.016 (0.734–1.408)	59/95	0.569 (0.354–0.915)	
Q3	174/176	1.053 (0.759–1.461)	85/93	0.785 (0.499–1.234)	
Q4(high)	231/173	1.423 (1.036–1.956)	127/104	1.034 (0.672–1.592)	
	rs12934922:AA		rs12934922:AT+TT		
High quality protein		*P*_*trend*_ = 0.065		*P*_*trend*_ = 0.119	0.69
Q1(low)	203/213	1.00(ref)	65/79	1.00(ref)	
Q2	209/221	1.132 (0.844–1.519)	72/64	1.400 (0.838–2.339)	
Q3	203/214	1.180 (0.877–1.588)	78/67	1.610 (0.965–2.686)	
Q4(high)	224/213	1.321 (0.984–1.773)	67/75	1.470 (0.878–2.463)	
Fruits and vegetables		*P*_*trend*_ < 0.001		*P*_*trend*_ < 0.001	0.447
Q1(low)	426/214	1.00(ref)	156/73	1.00(ref)	
Q2	185/227	0.430 (0.326–0.567)	59/65	0.432 (0.266–0.701)	
Q3	135/210	0.309 (0.229–0.416)	38/74	0.263 (0.156–0.443)	
Q4(high)	93/210	0.224 (0.162–0.309)	29/73	0.216 (0.124–0.376)	
Cereals and meat		*P*_*trend*_ = 0.701		*P*_*trend*_ = 0.035	0.129
Q1(low)	206/216	1.00(ref)	77/74	1.00(ref)	
Q2	217/226	0.903 (0.674–1.210)	82/59	1.275 (0.767–2.118)	
Q3	200/212	0.864 (0.641–1.164)	68/77	0.836 (0.510–1.370)	
Q4(high)	216/207	0.953 (0.707–1.283)	55/75	0.624 (0.369–1.053)	
Frugal pattern		*P*_*trend*_ = 0.079		*P*_*trend*_ = 0.725	0.619
Q1(low)	179/217	1.00(ref)	72/66	1.00(ref)	
Q2	191/223	0.933 (0.689–1.263)	55/69	0.701 (0.412–1.193)	
Q3	1194/207	0.938 (0.691–1.273)	71/77	0.821 (0.492–1.368)	
Q4(high)	275/214	1.287 (0.960–1.727)	84/73	1.031 (0.627–1.695)	
	rs7501331:CC		rs7501331:CT + TT		
High quality protein		*P*_*trend*_ = 0.097		*P*_*trend*_ = 0.134	0.902
Q1(low)	180/181	1.00(ref)	77/89	1.00(ref)	
Q2	172/177	1.139 (0.826–1.571)	92/101	1.189 (0.751–1.883)	
Q3	182/168	1.323 (0.957–1.827)	81/95	1.089 (0.678–1.748)	
Q4(high)	173/170	1.269 (0.917–1.756)	103/103	1.483 (0.934–2.354)	
Fruits and vegetables		*P*_*trend*_ < 0.001		*P*_*trend*_ < 0.001	0.487
Q1(low)	359/173	1.00(ref)	191/101	1.00(ref)	
Q2	160/175	0.464 (0.341–0.630)	65/99	0.372 (0.241–0.574)	
Q3	115/182	0.291 (0.210–0.402)	51/86	0.332 (0.209–0.528)	
Q4(high)	73/166	0.212 (0.147–0.305)	46/102	0.274 (0.172–0.435)	
Cereals and meat		*P*_*trend*_ = 0.110		*P*_*trend*_ = 0.948	0.44
Q1(low)	169/177	1.00(ref)	100/102	1.00(ref)	
Q2	202/179	1.083 (0.786–1.494)	77/89	0.778 (0.493–1.229)	
Q3	176/166	1.005 (0.725–1.395)	84/104	0.694 (0.443–1.088)	
Q4(high)	160/174	0.769 (0.550–1.074)	92/93	1.032 (0.662–1.610)	
Frugal pattern		*P*_*trend*_ = 0.014		*P*_*trend*_ = 0.727	0.253
Q1(low)	150/180	1.00(ref)	88/92	1.00(ref)	
Q2	153/175	0.979 (0.700–1.370)	76/100	0.709 (0.446–1.127)	
Q3	180/170	1.172 (0.842–1.630)	71/95	0.651 (0.405–1.048)	
Q4(high)	224/171	1.433 (1.036–1.982)	118/101	1.055 (0.682–1.632)	

## Discussion

In this study, we did not observe any associations of SNPs in *BCMO1* with lung cancer or dietary pattern related to lung cancer in a case-control study of 2345 unrelated Fujian Han Chinese participants. Because of the lack of linkage disequilibrium, we could not construct a haplotype of the three examined SNPs. Our factor analysis yielded four dietary patterns based on traditional Fujian dietary habits. The results of our analysis of baseline characteristics and lung cancer risk suggest that a diet rich in fruits and vegetables may be protective against lung cancer and the “cereals/wheat and meat” pattern was associated with a reduced risk and the protective effects were more evident for squamous cell carcinoma and other histological types and among smokers. In contrast, the “Frugal pattern” pattern was associated with an increased risk and the harmful effects were more pronounced for smokers. Finally, for the first time, we found that the effects of the “high quality protein” pattern was further modified by rs6564851.

Because BC, which is ubiquitous in edible plants, and BC metabolites have important biological functions, BC is generally considered to be a health promoting compound. However, lung exposure to BC in Bcmo1^−/−^ mice has been reported to alter gene expression in a manner that augments the Gene Ontology terms “oncogenes”, “cell proliferation”, and “cell cycle”. BC has also been reported to have adverse effects on lung tissues in human subjects, including increasing the risk of lung cancer [21, 27, 28]. BC absorption and conversion into retinal is extremely variable across individuals, with as many 45% of the people being classified as low responders to dietary BC [29]. Two *BCMO1* coding-region SNPs examined in this study (rs12934922 and rs7501331) were shown previously to result in reduced BCMO1 catalytic activity, confirming that these variants at least contribute to a low-responder phenotype. In vitro biochemical characterization of a double mutant BCMO1 protein encoded by recombinant gene carrying bot the rs12934922 and rs7501331 SNPs indicated that the double mutation reduced catalytic activity of BCMO1 by 57% (*P* < 0.001) [25]. Meanwhile, the homozygous rs6564851 genotype of *BCMO1* has been reported to result in a 48% reduction in the catalytic activity of BCMO1 as reflected by in vivo plasma level data in adult female human volunteers [29].

We speculated that efficiency-reducing *BCMO1* SNPs would allow accumulation of BC in vivo, which may support uncontrolled proliferation of lung cells. Our hypothesis that the low BC➔retinal efficiency *BCMO1* variant genotypes would thus be associated with lung cancer risk was not supported by the present results. Although the sample size of the current study is not small, the association of *BCOM1* polymorphisms can be examined with a larger study with a more comprehensive genotyping on *BCOM1* gene. This study was the first, to our knowledge, to examine the relationship between these variants and lung cancer directly. A prior Italian genome-wide association study did reveal an association between rs6564851 and higher than average BC levels, but the authors expected it would nonetheless be associated with a lower risk of cancer [26]. In our study, we observed that the effects of the “high quality protein” pattern was further modified by rs6564851. It showed there may have been some genetic mechanisms need to explore.

Notably, this study had the strength of employing dietary pattern analysis, which can better reveal dietary habit interactions and health benefits than studies of isolated nutrients. The results of a recent meta-analysis suggest that a healthful dietary pattern (a.k.a. a prudent pattern)—characterized by a high intake of vegetables, fruits, white meat, fish, and whole-grain breads and a low intake of red meat, fatty foods, and refined grains—is associated with a reduced lung cancer risk, and thus provide evidence for favoring diet pattern shifts in the general population [19].

The patterns identified in this analysis were reflective of real-world consumption in the Fujian Han population rather than an ideal dietary pattern. A potential criticism of this approach is that the dietary pattern factors are dependent on the study population for their validity. Thus, a different set of patterns may emerge with a different study population, which limits the interpretive value of these dietary patterns. However, it is important to note that our high quality protein (seafood in majority), fruits and vegetables patterns are analogous to patterns that have emerged repeatedly in many studies that used factor analysis to study dietary patterns [30, 31].

Our association findings for four patterns are consistent with findings from previous studies on dietary pattern and lung cancer. Previous factor-analysis studies [15–17] have related healthful eating to a decreased risk of lung cancer, similar to findings obtained with index-based dietary patterns, supporting the current dietary guidance of increasing consumption of fruits, vegetables, whole grains, lean meats or meat alternatives, and low-fat dairy [11]. In addition, the Mediterranean dietary pattern was thought to be negatively related with risk of lung cancer, whereas a “Western” dietary pattern was found to be associated with lung cancer risk [13].

On the other hand, our study showed a positive relationship between frugal pattern and lung cancer risk, which has not, to our knowledge, been reported previously. The participants in our study with high scores on the frugal pattern showed with a lower income. In Fujian, poor people usually take dried sweet potato and salted vegetables as staple food. This pattern showed increased incidence of lung cancer that suggests that there may have been relationship between economics and lung cancer. However, the potential mechanisms linking strong adherence to frugal pattern with an increased risk of lung cancer are unknown.

Our study had several strengths and limitations. The strengths include our large sample size, which tends to reduce type II errors. Additionally, extensive information on lifestyle factors were collected to enable adjustment for confounding factors. Several potential limitations of the present study should also be considered. Firstly, there was a recruitment bias related to the retrospective case-control study design; however, the results did not appear to be seriously affected by this bias given the HWEs in the control group. Secondly, our study was subject to potential dietary intake recall bias and we do not use 3-day measuring method or other methods to validate for each of the dietary patterns. It exits potential bias on the findings. Nevertheless, the directions and magnitudes of the associations for our patterns were consistent with other prospective studies. Finally, we did not employ a food-frequency questionnaire (FFQ) and thus may have missed the opportunity to capture data on more types of foods. Further studies in large population-based cohorts by using a FFQ are warranted to identify the role of dietary habits in lung cancer in Fujian, China.

## Conclusions

In summary, our study adds to the growing evidence indicating that diet plays an important role in lung carcinogenesis, which is often assumed to be caused solely by smoking. In particular, our study suggests that a diet rich in fruits and vegetables may reduce lung cancer risk.

## Additional files

Additional file 1: **Table S1.** All rotated factor loadings (multiplied by 100) for the 4 factors from principal components analysis of all line items from the food-frequency questionnaire. (DOCX 17 kb)

Additional file 2: **Table S2.** Baseline characteristics by quartile (Q) of factor scores. (DOCX 33 kb)

## Abbreviations

BC: β-carotene
BMI: Body mass index
CI: Confidence interval
ETS: Environmental tobacco smoking
HWE: Weinberg equilibrium
SNPs: Single nucleotide morphisms
