# Supplementary material for: Dietary patterns, BCMO1 polymorphisms, and primary lung cancer risk in a Han Chinese population: a case-control study in Southeast China
Source: BMC Cancer. 2018 Apr 19;18:445. doi: 10.1186/s12885-018-4361-2 (PMC5909209; doi:10.1186/s12885-018-4361-2)
Supplement: Supplementary file 1 — Table S1. All rotated factor loadings (multiplied by 100) for the 4 factors from principal components analysis of all line items from the food-frequency questionnaire. (DOCX 17 kb) [file 12885_2018_4361_MOESM1_ESM.docx]

| **Table S1. All rotated factor loadings (multiplied by 100) for the 4 factors from principal components analysis of all line items from the food-frequency questionnaire** | | | | |
| --- | --- | --- | --- | --- |
| Food | Factor1 | Factor2 | Factor3 | Factor4 |
| kelp/Seaweed | 0.784 |  |  |  |
| seafood | 0.623 |  |  |  |
| egg | 0.526 |  |  |  |
| beans | 0.468 |  |  |  |
| fruit |  | 0.685 |  |  |
| milk |  | 0.596 |  |  |
| vegetables |  | 0.594 |  |  |
| cereals/wheat |  |  | 0.735 |  |
| meat(pork/beef/lamb/poultry) |  |  | 0.549 |  |
| sweet potato |  |  |  | 0.594 |
| salted vegetables |  |  |  | 0.577 |
