# Supplementary material for: Dietary patterns, BCMO1 polymorphisms, and primary lung cancer risk in a Han Chinese population: a case-control study in Southeast China
Source: BMC Cancer. 2018 Apr 19;18:445. doi: 10.1186/s12885-018-4361-2 (PMC5909209; doi:10.1186/s12885-018-4361-2)
Supplement: Supplementary file 2 — Table S2. Baseline characteristics by quartile (Q) of factor scores. (DOCX 33 kb) [file 12885_2018_4361_MOESM2_ESM.docx]

| **Table S2. Baseline characteristics by quartile (Q) of factor scores** | | | | | | | | | | | | | | | | |
| --- | --- | --- | --- | --- | --- | --- | --- | --- | --- | --- | --- | --- | --- | --- | --- | --- |
| Variables | High quality protein | | | | Fruits and vegetables | | | | Cereals and meat | | | | Frugal pattern | | | |
|  | Q1 | Q2 | Q3 | Q4 | Q1 | Q2 | Q3 | Q4 | Q1 | Q2 | Q3 | Q4 | Q1 | Q2 | Q3 | Q4 |
| Age (yrs.) mean ± SD | 59.12± 11.51 | 59.89 ± 10.54 | 58.11 ± 11.46 | 57.84 ± 10.56† | 58.60±11.12 | 59.16±10.44 | 58.98±11.34 | 58.18±11.338 | 60.70±10.93 | 59.84±10.83 | 57.81±11.07 | 56.47±10.88† | 57.52±11.66 | 59.37±11.17 | 59.42±11.25 | 58.64±10.14 |
| < 50 | 121 (21.2) | 96 (16.4) | 138 (23.5) | 132 (22.0) | 191 (21.3) | 100 (18.0) | 98 (20.8) | 98 (23.3) | 91 (15.5) | 116 (19.0) | 130 (22.5) | 150 (26.2) | 144 (25.8) | 111 (20.1) | 104 (18.3) | 128 (19.2) |
| 51-69 | 341 (59.7) | 377 (64.2) | 353 (60.1) | 384 (64.0) | 556 (62.0) | 368 (66.2) | 282 (59.7) | 249 (59.3) | 360 (61.3) | 371 (60.9) | 369 (64.0) | 355 (62.1) | 321 (57.5) | 335 (60.8) | 354 (62..4) | 445 (66.5) |
| ≥ 70 | 109 (19.1) | 114 (19.4) | 96 (16.4) | 84 (14.0)* | 150 (16.7) | 88 (15.8) | 92 (19.5) | 73 (17.4) | 136 (23.2) | 122 (20.1) | 78 (13.5) | 67 (11.7)* | 93 (16.7) | 105 (19.1) | 109 (19.3) | 96 (14.3) |
| Income(yuan/month) |  |  |  |  |  |  |  |  |  |  |  |  |  |  |  |  |
| ≤3500 | 411 (72) | 410 (69.8) | 403 (68.7) | 409 (68.2) | 594 (66.2) | 392 (70.5) | 352 (74.6) | 295 (70.2) | 390 (66.4) | 422 (69.3) | 419 (72.6) | 402 (70.3) | 374 (67) | 373 (67.7) | 392 (69.1) | 494 (73.8) |
| ≥3500 | 160 (28) | 177 (30.2) | 184 (31.3) | 191 (31.8) | 303 (33.8) | 164 (29.5) | 120 (25.4) | 125 (29.8)* | 197 (33.6) | 187 (30.7) | 158 (27.4) | 170 (29.7) | 184 (33) | 178 (32.3) | 175 (30.9) | 175 (26.2)* |
| Gender |  |  |  |  |  |  |  |  |  |  |  |  |  |  |  |  |
| male | 409 (71.6) | 425 (72.4) | 427 (72.7) | 419 (69.8) | 624 (69.6) | 411 (73.9) | 345 (73.1) | 300 (71.4) | 451 (76.8) | 466 (76.5) | 394 (68.3) | 369 (64.5) | 386 (69.2) | 391 (71) | 419 (73.9) | 484 (72.3) |
| female | 162 (28.4) | 162 (27.6) | 160 (27.3) | 181 (30.2) | 273 (30.4) | 145 (26.1) | 127 (26.9) | 120 (28.6) | 136 (23.2) | 143 (23.5) | 183 (31.7) | 203 (35.5)* | 172 (30.8) | 160 (29) | 148 (26.1) | 185 (27.7) |
| Education |  |  |  |  |  |  |  |  |  |  |  |  |  |  |  |  |
| illiteracy | 92 (16.1) | 81 (13.8) | 79 (13.5) | 66 (11) | 152 (16.9) | 73 (13.1) | 59 (12.5) | 34 (8.1) | 75 (12.8) | 81 (13.3) | 86 (14.9) | 76 (13.3) | 51 (9.1) | 76 (13.8) | 82 (14.5) | 109 (16.3) |
| middle school and below | 331 (58) | 345 (58.8) | 332 (56.6) | 325 (54.2) | 544 (60.6) | 325 (58.5) | 253 (53.6) | 211 (50.2) | 311 (53) | 355 (58.3) | 329 (57) | 338 (59.1) | 302 (54.1) | 327 (59.3) | 319 (56.3) | 385 (57.5) |
| high school and above | 148 (25.9) | 161 (27.4) | 176 (30) | 209 (34.8)* | 201 (22.4) | 158 (28.4) | 160 (33.9) | 175 (41.7)* | 201 (34.2) | 173 (28.4) | 162 (28.1) | 158 (27.6) | 205 (36.7) | 148 (26.9) | 166 (29.3) | 175 (26.2)* |
| Marital status |  |  |  |  |  |  |  |  |  |  |  |  |  |  |  |  |
| married | 529 (92.6) | 545 (92.8) | 554 (94.4) | 565 (94.2) | 841 (93.8) | 521 (93.7) | 442 (93.6) | 389 (92.6) | 544 (92.7) | 569 (93.4) | 545 (94.5) | 535 (93.5) | 526 (94.3) | 512 (92.9) | 527 (92.9) | 628 (93.9) |
| single | 42 (7.4) | 42 (7.2) | 33 (5.6) | 35 (5.8) | 56 (6.2) | 35 (6.3) | 30 (6.4) | 31 (7.4) | 43 (7.3) | 40 (6.6) | 32 (5.5) | 37 (6.5) | 32 (5.7) | 39 (7.1) | 40 (7.1) | 41 (6.1) |
| Occupation |  |  |  |  |  |  |  |  |  |  |  |  |  |  |  |  |
| worker | 134 (23.5) | 149 (25.4) | 134 (22.8) | 146 (24.3) | 231 (25.8) | 134 (24.1) | 109 (23.1) | 89 (21.2) | 141 (24) | 150 (24.6) | 129 (22.4) | 143 (25) | 133 (23.8) | 138 (25) | 137 (24.2) | 155 (23.2) |
| farmer | 175 (30.6) | 153 (26.1) | 129 (22) | 121 (20.2) | 241 (26.9) | 153 (27.5) | 108 (22.9) | 76 (18.1) | 123 (21) | 156 (25.6) | 142 (24.6) | 157 (27.4) | 108 (19.4) | 124 (22.5) | 144 (25.4) | 202 (30.2) |
| enterprises and employees | 158 (27.7) | 175 (29.8) | 205 (34.9) | 216 (36) | 234 (26.1) | 171 (30.8) | 165 (35) | 184 (43.8) | 225 (38.3) | 185 (30.4) | 186 (32.2) | 158 (27.6) | 213 (38.2) | 162 (29.4) | 182 (32.1) | 197 (29.4) |
| cook | 4 (0.7) | 5 (0.9) | 10 (1.7) | 8 (1.3) | 10 (1.1) | 5 (0.9) | 9 (1.9) | 3 (0.7) | 6 (1) | 9 (1.5) | 5 (0.9) | 7 (1.2) | 6 (1.1) | 11 (2) | 6 (1.1) | 4 (0.6) |
| others | 100 (17.5) | 105 (17.9) | 109 (18.6) | 109 (18.2) | 181 (20.2) | 93 (16.7) | 81 (17.2) | 68 (16.2) | 92 (15.7) | 109 (17.9) | 115 (19.9) | 107 (18.7) | 98 (17.6) | 116 (21.1) | 98 (17.3) | 111 (16.6) |
| Family history of lung cancer |  |  |  |  |  |  |  |  |  |  |  |  |  |  |  |  |
| no | 481 (84.2) | 484 (82.5) | 489 (83.3) | 493 (82.2) | 740 (82.5) | 458 (82.4) | 399 (84.5) | 350 (83.3) | 508 (86.5) | 518 (85.1) | 476 (82.5) | 445 (77.8) | 469 (84.1) | 467 (84.8) | 463 (81.7) | 548 (81.9) |
| yes | 90 (15.8) | 103 (17.5) | 98 (16.7) | 107 (17.8) | 157 (17.5) | 98 (17.6) | 73 (15.5) | 70 (16.7) | 79 (13.5) | 91 (14.9) | 101 (17.5) | 127 (22.2)* | 89 (15.9) | 84 (15.2) | 104 (18.3) | 121 (18.1) |
| History of lung diseases |  |  |  |  |  |  |  |  |  |  |  |  |  |  |  |  |
| no | 515 (90.2) | 520 (88.6) | 523 (89.1) | 531 (88.5) | 794 (88.5) | 500 (89.9) | 425 (90) | 370 (88.1) | 513 (87.4) | 542 (89) | 517 (89.6) | 517 (90.4) | 498 (89.2) | 502 (91.1) | 497 (87.7) | 592 (88.5) |
| yes | 56 (9.8) | 67 (11.4) | 64 (10.9) | 69 (11.5) | 103 (11.5) | 56 (10.1) | 47 (10) | 50 (11.9) | 74 (12.6) | 67 (11) | 60 (10.4) | 55 (9.6) | 60 (10.8) | 49 (8.9) | 70 (12.3) | 77 (11.5) |
| BMI (kg/m^2^) |  |  |  |  |  |  |  |  |  |  |  |  |  |  |  |  |
| 18.5-23.9 | 346 (60.8) | 332 (57) | 339 (57.9) | 341 (57) | 538 (60.2) | 314 (56.8) | 264 (56.3) | 242 (57.9) | 345 (59) | 367 (60.8) | 332 (57.7) | 314 (55.1) | 311 (56.1) | 323 (58.7) | 334 (59.3) | 390 (58.5) |
| <18.5 | 50 (8.8) | 51 (8.8) | 39 (6.7) | 44 (7.4) | 91 (10.2) | 37 (6.7) | 32 (6.8) | 24 (5.7) | 40 (6.8) | 41 (6.8) | 49 (8.5) | 54 (9.5) | 45 (8.1) | 47 (8.5) | 43 (7.6) | 49 (7.3) |
| ≥24 | 173 (30.4) | 199 (34.2) | 207 (35.4) | 213 (35.6) | 265 (29.6) | 202 (36.5) | 173 (36.9) | 152 (36.4) | 200 (34.2) | 196 (32.5) | 194 (33.7) | 202 (35.4) | 198 (35.7) | 180 (32.7) | 186 (33) | 228 (34.2) |
| Tea |  |  |  |  |  |  |  |  |  |  |  |  |  |  |  |  |
| no | 297 (52) | 317 (54) | 268 (45.7) | 272 (45.3) | 472 (52.6) | 282 (50.7) | 214 (45.3) | 186 (44.3) | 271 (46.2) | 277 (45.5) | 307 (53.2) | 299 (52.3) | 274 (49.1) | 285 (51.7) | 280 (49.4) | 315 (47.1) |
| yes | 274 (48) | 270 (46) | 319 (54.3) | 328 (54.7)* | 425 (47.4) | 274 (49.3) | 258 (54.7) | 234 (55.7)* | 316 (53.8) | 332 (54.5) | 270 (46.8) | 273 (47.7)* | 284 (50.9) | 266 (48.3) | 287 (50.6) | 354 (52.9) |
| Alcohol |  |  |  |  |  |  |  |  |  |  |  |  |  |  |  |  |
| no | 448 (78.5) | 440 (75) | 447 (76.1) | 462 (77) | 674 (75.1) | 431 (77.5) | 363 (76.9) | 329 (78.3) | 447 (76.1) | 454 (74.5) | 454 (78.7) | 442 (77.3) | 434 (77.8) | 410 (74.4) | 439 (77.4) | 514 (76.8) |
| yes | 123 (21.5) | 147 (25) | 140 (23.9) | 138 (23) | 223 (24.9) | 125 (22.5) | 109 (23.1) | 91 (21.7) | 140 (23.9) | 155 (25.5) | 123 (21.3) | 130 (22.7) | 124 (22.2) | 141 (25.6) | 128 (22.6) | 155 (23.2) |
| Smoking |  |  |  |  |  |  |  |  |  |  |  |  |  |  |  |  |
| no | 276 (48.3) | 279 (47.5) | 277 (47.2) | 286 (47.7) | 393 (43.8) | 253 (45.5) | 237 (50.2) | 235 (56) | 274 (46.7) | 252 (41.4) | 288 (49.9) | 304 (53.1) | 280 (50.2) | 270 (49) | 267 (47.1) | 301 (45) |
| yes | 295 (51.7) | 308 (52.5) | 310 (52.8) | 314 (52.3) | 504 (56.2) | 303 (54.5) | 235 (49.8) | 185 (44)* | 313 (53.3) | 357 (58.6) | 289 (50.1) | 268 (46.9)* | 278 (49.8) | 281 (51) | 300 (52.9) | 368 (55) |
| ETS |  |  |  |  |  |  |  |  |  |  |  |  |  |  |  |  |
| no | 216 (37.8) | 259 (44.1) | 246 (41.9) | 274 (45.7) | 344 (38.4) | 256 (46) | 203 (43) | 192 (45.7) | 268 (45.7) | 271 (44.5) | 231 (40) | 225 (39.3) | 253 (45.3) | 251 (45.6) | 235 (41.4) | 256 (38.3) |
| yes | 355 (62.2) | 328 (55.9) | 341 (58.1) | 326 (54.3)* | 553 (61.6) | 300 (54) | 269 (57) | 228 (54.3)* | 319 (54.3) | 338 (55.5) | 346 (60) | 347 (60.7)* | 305 (54.7) | 300 (54.4) | 332 (58.6) | 413 (61.7)* |
| Histology |  |  |  |  |  |  |  |  |  |  |  |  |  |  |  |  |
| Adenocarcinoma | 126 (45.5) | 130 (44.8) | 142 (48.6) | 153 (50) | 283 (46.9) | 121 (46.4) | 85 (48.3) | 62 (49.6) | 117 (39.9) | 143 (45.5) | 127 (45.4) | 164 (59) | 126 (47.7) | 117 (45.9) | 118 (43.4) | 190 (50.8) |
| Squamous cell carcinoma | 74 (26.7) | 85 (29.3) | 88 (30.1) | 77 (25.2) | 160 (26.5) | 79 (30.3) | 51 (29) | 34 (27.2) | 92 (31.4) | 93 (29.6) | 83 (29.6) | 56 (20.1) | 75 (28.4) | 72 (28.2) | 81 (29.8) | 96 (25.7) |
| Others | 77 (27.8) | 75 (25.9) | 62 (21.2) | 76 (24.8) | 160 (26.5) | 61 (23.4) | 40 (22.7) | 29 (23.2) | 84 (28.7) | 78 (24.8) | 70 (25) | 58 (20.9)* | 63 (23.9) | 66 (25.9) | 73 (26.8) | 88 (23.5) |
| † P for correlation <0.05 for each factor | | | | | | | | | | | | | | | | |
| * P for liner trend <0.05 for each factor | | | | | | | | | | | | | | | | |
